# Deoxyschizandrin Inhibits the Proliferation, Migration, and Invasion of Bladder Cancer Cells through ALOX5 Regulating PI3K-AKT Signaling Pathway

**DOI:** 10.1155/2022/3079823

**Published:** 2022-05-25

**Authors:** Baojin Chi, Yao Sun, Jintao Zhao, Yugang Guo

**Affiliations:** ^1^Department of Urology, The First Affiliated Hospital of Jiamusi University, Heilongjiang 154007, China; ^2^Department of Vascular Surgery, The First Affiliated Hospital of Jiamusi University, Heilongjiang 154007, China; ^3^Department of Gastroenterology, The First Affiliated Hospital of Jiamusi University, Heilongjiang 154007, China

## Abstract

**Objective:**

Deoxyschizandrin has a significant inhibitory effect on a variety of tumor cells. However, the effect of Deoxyschizandrin on bladder cancer cells and its mechanism are still unclear.

**Methods:**

Bladder cancer cells were treated with different concentrations of Deoxyschizandrin for 24 h, 48 h, and 72 h. The inhibition rate of cell proliferation was detected by CCK-8 assay. The changes of cell migration and invasion were detected by wound healing and Transwell assay. Based on the structure of Deoxyschizandrin, the protein targets of Deoxyschizandrin were predicted by bioinformatics database and verified by RNA and protein. Then, the expressions of ALOX5 and PI3K-AKT signaling pathway proteins were detected by Western blot in bladder cancer cells treated with Deoxyschizandrin.

**Result:**

Deoxyschizandrin inhibited the proliferation, migration, and invasion of bladder cancer cells in a time- and concentration-dependent manner. Bioinformatics analysis showed that Deoxyschizandrin had 100 protein targets; among them, the score of ALOX5 was the highest, and the mRNA and protein levels of ALOX5 decreased after treatment with different concentrations of Deoxyschizandrin. Western blot results showed that compared with the control group, Deoxyschizandrin could significantly reduce the expression of p-PI3K and p-AKT, and overexpression of ALOX5 could significantly enhance the expression of p-PI3K and p-AKT. Compared with Deoxyschizandrin or overexpression of ALOX5, the expression of p-PI3K and p-AKT of Deoxyschizandrin combined with overexpression of ALOX5 recovered.

**Conclusion:**

Deoxyschizandrin inhibits the proliferation, migration, and invasion of bladder cancer cells through ALOX5 regulating PI3K-AKT signaling pathway.

## 1. Introduction

Bladder cancer has been a common malignant tumor worldwide. The International Cancer Research Center shows that the number of new cases of bladder cancer in 2020 is about 573,278 and the number of deaths is about 212,536 in the world. The incidence rate of bladder cancer is sixth in male malignant tumors and ninth in mortality [1]. Bladder cancer can be divided into nonmuscle invasive bladder cancer (NMIBC) and muscle invasive bladder cancer (MIBC). Nonmuscle invasive bladder cancer accounts for 75% of bladder cancer [2]. Therefore, it is particularly important to explore the molecular mechanism of the invasion and metastasis of the bladder cancer and to study the drugs that inhibit the metastasis ability of the bladder cancer. Therefore, it is of great significance to find drugs for the treatment of bladder cancer.

Deoxyschizandrin schizandrin is a lignan compound in the extract of dried and mature fruit of Schisandra chinensis (Turcz.) Baill, which is a Magnoliaceae plant. Deoxyschizandrin is one of the effective components of Schisandra chinensis [3]. It has vasodilating, immunosuppressive, anti-inflammatory, and antiviral activities [4].

A large number of experiments have also verified that Deoxyschizandrin plays a significant role in the process of antitumor treatment. It can not only significantly inhibit the growth and induce apoptosis of a variety of tumor cells but also inhibit EMT and tumor angiogenesis of various tumors through a variety of signal pathways, so as to inhibit tumor invasion and metastasis, such as breast cancer and thyroid cancer, glioma, pancreatic cancer, liver cancer, and ovarian cancer [5–11]. However, the effect of Deoxyschizandrin on bladder cancer cells and its mechanism are still unclear. So the effects of Deoxyschizandrin on the proliferation, migration, invasion, and apoptosis of bladder cancer cells were investigated, so as to provide a new theoretical and experimental basis for the application of schisandrin A in the clinical treatment of bladder cancer.

ALOX5 (arachidonic acid 5-lipoxygenase) is a key enzyme in arachidonic acid metabolism. ALOX5 gene is located on chromosome 10q11.2, with a length of 7 188 kb. The expression is low in the physiological state, upregulated in pathological situations, and is related to the occurrence and development of the disease. It is related to the prognosis and progression of tumors [12–15], which plays an important role in malignant biological behavior of cancer cells. In a variety of solid tumors, the abnormal expression or overexpression of ALOX5 is related to tumor cell proliferation, differentiation, invasion, metastasis, inhibition of apoptosis, and so on. ALOX5 was found to promote tumorigenesis through the regulation of p53 [16–19].

In this experiment, we found that Deoxyschizandrin can inhibit proliferation, invasion, metastasis, and clone formation in vitro and promote apoptosis in bladder cancer cells. Further, we also revealed the molecular mechanism of Deoxyschizandrin in bladder cancer cells, which laid a theoretical foundation for the application of Deoxyschizandrin in bladder cancer.

## 2. Materials and Methods

### 2.1. Cell Culture

HT1376 and J82 cells were purchased from the Cell Collection Committee of the Chinese Academy of Sciences (Shanghai, China). HT1376 and J82 cells were cultured in RPMI-1640 medium (Gibco, USA) containing 10% fetal bovine serum (FBS) (Gibco, USA) at 37°C, 5% CO_2_. When the degree of cell fusion is 75%-80%, the cells were digested and passaged with 0.25% trypsin solution. The logarithmic growth phase cells in good condition were selected for subsequent experiments.

### 2.2. Cell Transfection

The cell density was adjusted to 1 × 10^5^ cells/mL. Then, 2 mL cell suspension was added and inoculated into 6-well plate and cultured at 37°C, 5% CO_2_ for 48 h. Subsequently, the ALOX5 overexpression vector and negative control (NC) vector were transfected into HT1376 and J82 cells. The transfection method was referred to the instructions of Lipofectamine 3000 transfection reagent (Invitrogen, USA). After 48 hours of transfection, total RNA and protein were extracted from HT1376 and J82 cells, and the expression of ALOX5 mRNA and protein was detected by RT-PCR and Western blot, respectively.

### 2.3. CCK-8 Assay

The cell density was adjusted to 2 × 10^4^ cells/mL. Then, 0.1 mL cell suspension was added and inoculated into 96-well plate and cultured at 37°C, 5% CO_2_ for 12 h. The cells were treated with 0.5, 5, 10, 50, and 100 *μ*mol/L Deoxyschizandrin (Med Chem Express, USA), and the control group was added with equal volume PBS for 24 h, 48 h, and 72 h, respectively. Then, 10 *μ*L CCK-8 reagent (Dojindo, Japan) was added and incubated for 2 h. The absorbance was detected by the BioTek (Winooski, USA) microplate spectrophotometer at 450 nm and repeat 3 times for each group.

### 2.4. RNA Isolation and Real-Time PCR

Total RNA was extracted by TRIzol reagent (Invitrogen, CA, USA) and then reverse transcribed into cDNA by the TaKaRa system (Takara, Dalian, China). According to the instructions of RT-PCR kit (Thermo Fisher, USA), the relative expression was calculated by formula 2^−*ΔΔ*Ct^.

### 2.5. Wound Healing

The cell density was adjusted to 5 × 10^5^ cells/mL. Then, 1 mL cell suspension was added and inoculated into 6-well plate and cultured at 37°C, 5% CO_2_ for 48 h. The cells are covered with 6-well plates, using a 200 *μ*L pipette tip to make a “-” one scratch along the bottom. After 0.5, 5, and 10 *μ*mol/L Deoxyschizandrin treatment of the cells for 24 h, the scratch separation was observed, measured, and photographed, and the cell migration inhibition rate was calculated.

### 2.6. Transwell Assay

The cell density was adjusted to 1 × 10^5^ cells/mL by serum free medium. Then, 0.1 mL cell suspension was added and inoculated onto the upper chambers and the lower chambers and was added 0.5 mL containing 10% FBS medium. After 0.5, 5, and 10 *μ*mol/L Deoxyschizandrin treatment of the cells for 48 h, the culture medium was discarded, and the upper chambers were washed with PBS for three times, then fixed with 4% paraformaldehyde solution, and stained with crystal violet for 15 min. The number of invasive cells was calculated by light microscope (Nikon, Japan).

### 2.7. In Vitro Tumor Xenografts

The cell density was adjusted to 1 × 10^7^ cells/mL by serum free medium. Then, 0.2 mL cell suspension was added into the upper back of nude mice. Subsequently, 25 mg/kg Deoxyschizandrin or PBS was injected into nude mice. The tumor volume of nude mice was measured every 7 days for 4 weeks, and the tumor volume was calculated by formula 1/2 × L × W^2^. This study had been approved by the Committee on the Ethics of Animal Experiments of the First Affiliated Hospital of Jiamusi University. The ethics committee approval number of our study is 2018031.

### 2.8. Bioinformatics Prediction of Protein Targets of Deoxyschizandrin

The three-dimensional structure file of Deoxyschizandrin was downloaded from the PubChem compound database (https://www.ncbi.nlm.nih.gov/pccompound); then, we uploaded the structure file of Deoxyschizandrin into SwissTargetPrediction database (http://www.swisstargetprediction.ch/) to obtain the protein target of Deoxyschizandrin.

### 2.9. Western Blot Assay

The cell density was adjusted to 1 × 10^6^ cells/mL. Then, 1 mL cell suspension was added and inoculated into 6-well plate and cultured at 37°C, 5% CO_2_ for 24 h. The cells were treated with 5, 10, and 50 *μ*mol/L Deoxyschizandrin, and the control group was added with equal volume PBS for 24 h. The cells were lysed with RIPA reagent containing 1% PMSF and centrifuged 12 000 r/min at 4°C for 15 min. The protein concentration was quantified by BCA assay (Thermo Scientific, USA). Then, 30 *μ*g protein samples were loaded, electrophoretic, transferred, and blocked. The primary antibodies (1 : 1 000) were added and incubated overnight at 4°C and washed by TBST three times. The corresponding secondary antibodies were added and incubated at room temperature for 120 min and washed by TBST three times. The exposure solution was added dropwise in a dark room for exposure development, and the protein target band was analyzed with ImageJ software. The experiment was repeated three times.

### 2.10. Statistical Analysis

Statistical analysis was performed using GraphPad Prism 6.0 statistical software. Cell proliferation rate, cell migration, cell invasion, tumor formation in vitro, RT-PCR, cell apoptosis rate, and other measurement data are shown as $\overset{¯}{x}\pm s$, and *t* test is used for comparison between two groups. *p* < 0.05 was considered statistically significant.

## 3. Results

### 3.1. Deoxyschizandrin Inhibits the Proliferation of Bladder Cancer Cell Line

**Figure 1:**
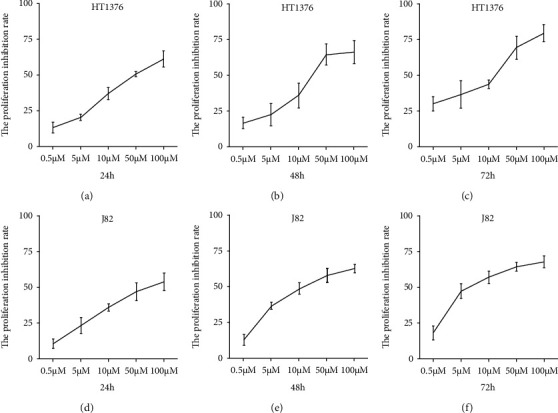
Deoxyschizandrin inhibits the proliferation of bladder cancer cell lines. (a–f) CCK-8 assay showed that Deoxyschizandrin inhibits cell proliferation in HT1376 and J82 cells. Data represent mean ± SD. ^∗^*p* < 0.05 compared with negative control.

At first, two kinds of bladder cancer cells were treated with different concentration gradients of Deoxyschizandrin (0.5, 5, 10, 50, and 100 *μ*mol/L) and different time (24 h, 48 h, and 72 h). Then, CCK-8 assay was used to analyze the growth and proliferation of the two kinds of cells (HT1376 and J82). At 24 h, 48 h, and 72 h, Deoxyschizandrin had a significant inhibitory effect on the growth of bladder cancer cells (HT1376 and J82), which increased with the increase of concentration in a dose-dependent manner (Figures 1(a)–1(f)).

### 3.2. Deoxyschizandrin Inhibits the Migration, Invasion, and In Vitro Tumorigenesis of Bladder Cancer Cell Lines

**Figure 2:**
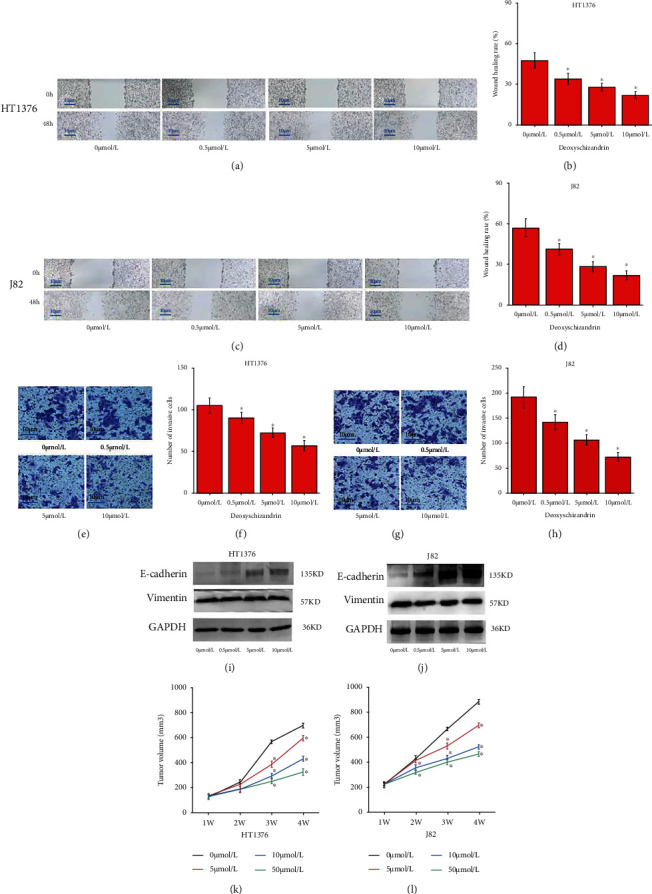
Deoxyschizandrin inhibits the migration, invasion, and in vitro tumorigenesis of bladder cancer cell lines. (a and b) Wound healing showed that Deoxyschizandrin significantly suppressed migration in HT1376 cells. (c and d) Wound healing showed that Deoxyschizandrin significantly suppressed migration in J82 cells. (e and f) Transwell assays showed that Deoxyschizandrin significantly suppressed invasion in HT1376 cells. (g and h) Transwell assays showed that Deoxyschizandrin significantly suppressed invasion in J82 cells. (i and j) The expression of E-cadherin, Vimentin protein level with Deoxyschizandrin treatment in HT1376 and J82 cells. (k and l) BALB/c nude mice injected into HT1376 and J82 cells had smaller volume tumor xenografts with Deoxyschizandrin treat. Data represent mean ± SD. ^∗^*p* < 0.05 compared with negative control.

In order to study the effect of Deoxyschizandrin on the migration ability of bladder cancer cells, according to the above experimental results, 0.5, 5, and 10 *μ*mol/L Deoxyschizandrin were used to treat bladder cancer cells. After 48 hours, the migration and invasion ability of bladder cancer cells (HT1376 and J82) were significantly reduced, which was significantly lower than that of the control group (Figures 2(a)–2(h)). WB revealed that E-cadherin was decreased after Deoxyschizandrin treatment (Figures 2(i) and 2(j)). Similar results were obtained in vitro tumorigenesis (Figures 2(k) and 2(l)).

### 3.3. Bioinformatics Prediction of Protein Targets of Deoxyschizandrin

**Figure 3:**
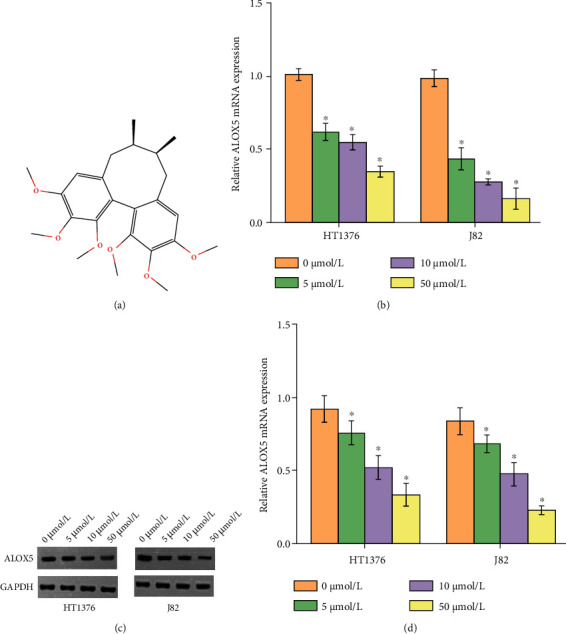
Deoxyschizandrin can regulate the expression of ALOX5 in BC cell. (a) The chemical structure of Deoxyschizandrin. (b) qRT-PCR analysis of mRNA expression levels of ALOX5 in HT1376 and J82 cells treated with Deoxyschizandrin or negative control. (c and d) Western blot analysis of protein expression levels of ALOX5 in HT1376 and J82 cells treated with Deoxyschizandrin or negative control. Data represent mean ± SD. ^∗^*p* < 0.05 compared with negative control.

Using the online database PubChem (https://pubchem.ncbi.nlm.nih.gov/) to download the chemical structure of Deoxyschizandrin (Figure 3(a)) and then using the online database SwissTargetPrediction (http://www.swisstargetprediction.ch/index.php), we predicted that there were 100 protein targets of Deoxyschizandrin. It was found that the protein target with the highest degree score was ALOX5. In order to study whether Deoxyschizandrin can regulate the expression of ALOX5, bladder cancer cells were treated with 5, 10, and 50 *μ*mol/L Deoxyschizandrin. After 48 hours, the expressions of ALOX5 mRNA and protein were decreased with the concentration of Deoxyschizandrin (Figures 3(b)–3(d)).

### 3.4. Deoxyschizandrin Inhibits the Proliferation, Migration, and Invasion of Bladder Cancer Cells by Downregulating ALOX5 and Affecting the Downstream PI3K-Akt Signaling Pathway

**Figure 4:**
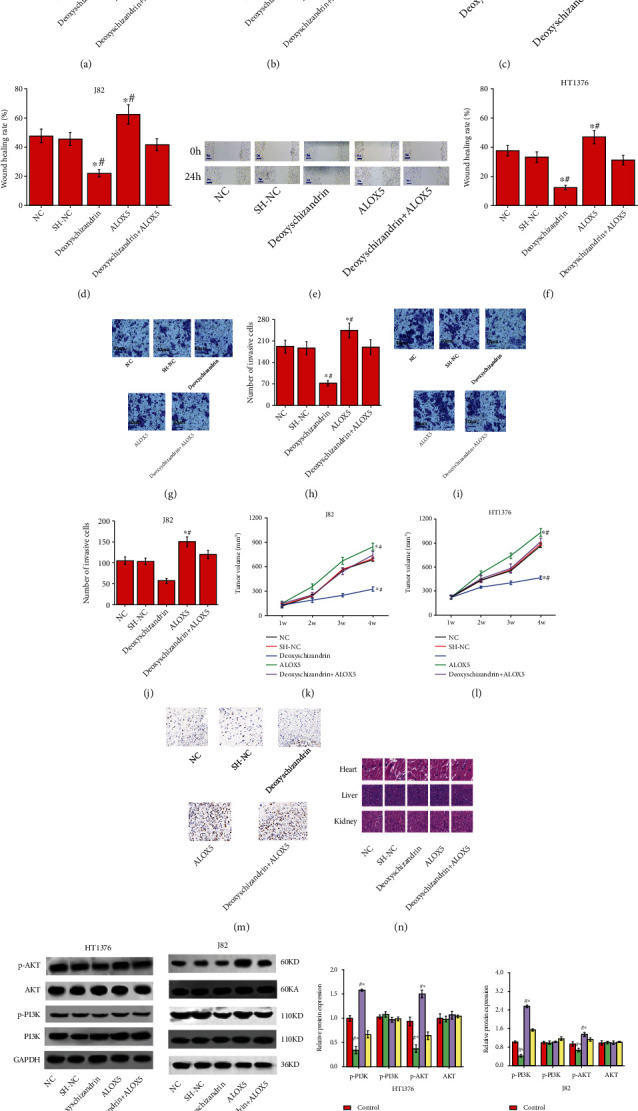
Deoxyschizandrin inhibits the proliferation, migration, and invasion of bladder cancer cells by downregulating ALOX5 and affecting the downstream PI3K-Akt signaling pathway. (a and b) CCK-8 assay showed that negative control (NC) or sh-NC or Deoxyschizandrin or overexpression-ALOX5 or Deoxyschizandrin+overexpression-ALOX5 could regulate HT1376 cells' proliferation. (c and d) Wound healing assay showed that negative control (NC) or sh-NC or Deoxyschizandrin or overexpression-ALOX5 or Deoxyschizandrin+overexpression-ALOX5 could regulate HT1376 cells' migration. (e and f) Wound healing assay showed that negative control (NC) or sh-NC or Deoxyschizandrin or overexpression-ALOX5 or Deoxyschizandrin+overexpression-ALOX5 could regulate J82 cells' migration. (g and h) Transwell assay showed that negative control (NC) or sh-NC or Deoxyschizandrin or overexpression-ALOX5 or Deoxyschizandrin+overexpression-ALOX5 could regulate HT1376 cells' invasion. (i and j) Transwell assay showed that negative control (NC) or sh-NC or Deoxyschizandrin or overexpression-ALOX5 or Deoxyschizandrin+overexpression-ALOX5 could regulate J82 cells' invasion. (k and l) BALB/c nude mice injected into HT1376 cells had smaller volume tumor xenografts with negative control (NC) or sh-NC or Deoxyschizandrin or overexpression-ALOX5 or Deoxyschizandrin+overexpression-ALOX5 treatment. (m) Ki-67 assay showed that negative control (NC) or sh-NC or Deoxyschizandrin or overexpression-ALOX5 or Deoxyschizandrin+overexpression-ALOX5 treatment by BALB/c nude mice injected into HT1376 cells. (n) HE stain revealed that negative control (NC) or sh-NC or Deoxyschizandrin or overexpression-ALOX5 or Deoxyschizandrin+overexpression-ALOX5 treatment by BALB/c nude mice injected into HT1376 cells. (o and p) Western blot analysis; the expression levels of AKT, PI3K, p-AKT, and P-PI3K were treated negative control (NC) or sh-NC or Deoxyschizandrin or overexpression-ALOX5 or Deoxyschizandrin+overexpression-ALOX5 in HT1376 and J82 cells. Data represent mean ± SD. ^∗^*p* < 0.05 and ^#^*p* < 0.05 compared with Deoxyschizandrin+overexpression-ALOX5.

The results of CCK-8, wound healing, and Transwell assay showed that compared with the control group, the proliferation, migration, and invasion ability of bladder cancer cells were enhanced after overexpression of ALOX5, while the proliferation, migration, and invasion ability of bladder cancer cells were decreased after overexpression of ALOX with Deoxyschizandrin (Figures 4(a)–4(j)). Similar results were obtained in vitro tumorigenesis and Ki-67 (Figures 4(k)–4(m)). HE results the toxicity of the liver and kidney, and Deoxyschizandrin displayed no obvious cytotoxicity in mice (Figure 4(n)).

Western blot results showed that overexpression of ALOX5 could increase the expression of p-PI3K and p-AKT protein compared with the control group. The expression of p-PI3K and p-AKT protein was decreased after treatment with Deoxyschizandrin, while the expression of p-PI3K and p-AKT protein was recovered after treatment with Deoxyschizandrin and overexpression of ALOX5 group (Figures 4(o) and 4(p)). These results indicated that Deoxyschizandrin can inhibit the proliferation, migration, and invasion of bladder cancer cells by ALOX5 and regulating the downstream PI3K-Akt signaling pathway.

## 4. Discussion

Bladder cancer is a common tumor of the urinary system. Incidence rate and mortality rate have been increasing year by year. 10%-30% of bladder cancer will progress to muscle invasive bladder cancer with high invasion, rapid progression, and metastasis, and the prognosis is often poor [20–24]. Therefore, it is very important to study the antitumor drugs for bladder cancer. Chemotherapy is an important part of the treatment plan, especially after bladder cancer surgery. At present, more and more drug resistance is produced in the occurrence and development of tumor, which brings new challenges to the treatment [25–27]. Therefore, it is an important strategy to find new drugs or treatment methods and reduce the distant metastasis of bladder cancer.

In recent years, the potential value of Chinese medicine extracts in China has increased. More and more attention has been paid to the value of traditional Chinese medicine extracts in the treatment of bladder cancer. Currently, a variety of traditional Chinese medicines and their active ingredients have demonstrated good activity against bladder cancer. Schisandra chinensis has reported antitumor effect, and its antitumor effect is mainly concentrated in lignans and polysaccharides. Deoxyschizandrin is one of the main components of lignans.

In recent years, more and more studies have shown that schisandrin A has significant and extensive antitumor activity, and the research on its molecular mechanism has gradually deepened. Schisandrin A can effectively inhibit tumor cell proliferation, induce tumor cell apoptosis, reverse tumor cell chemoresistance, inhibit tumor cell invasion and metastasis, and cooperate with anticancer drugs to enhance efficacy and chemosensitivity. The results showed that different concentrations of Deoxyschizandrin could significantly inhibit the growth, migration, invasion, and in vitro tumorigenesis and promote apoptosis of bladder cancer cells in a concentration dependent manner. It is suggested that Deoxyschizandrin has potential medicinal value in the treatment of bladder cancer. At present, the antitumor research of SCH A is mainly focused on the cell level. Most of the research on the synergistic effect produced by the combination of Deoxyschizandrin and various chemotherapeutic drugs are limited to cell experiments and a small number of animal experiments. There are few related clinical trials. More research is still needed to prove the effect of Deoxyschizandrin's antitumor effect in human body.

PI3K/AKT signaling pathway, as an oncogene signaling pathway, plays an important role in cell growth, proliferation, survival, transcription, and protein synthesis [28–32]. In our study, Deoxyschizandrin inhibits PI3K/AKT signaling pathway through ALOX5, which is closely related to inhibiting cell proliferation and promoting apoptosis. Western blot analysis showed that Deoxyschizandrin could inhibit the expression of p-PI3K and p-AKT.

## 5. Conclusion

The results show that Deoxyschizandrin can inhibit the proliferation and migration of bladder cancer cells in vivo and in vitro and promote cell apoptosis through PI3K/AKT signaling pathway by ALOX5, indicating that Deoxyschizandrin may be used in the treatment of bladder cancer, but more cell line application studies and clinical studies are needed to verify it.

## Data Availability

The data used to support the findings of this study are included in the article. Further inquiries can be directed to the corresponding author.
